# Cr-Based Sputtered Decorative Coatings for Automotive Industry

**DOI:** 10.3390/ma14195527

**Published:** 2021-09-24

**Authors:** Edgar Carneiro, Nuno M. G. Parreira, Todor Vuchkov, Albano Cavaleiro, Jorge Ferreira, Martin Andritschky, Sandra Carvalho

**Affiliations:** 1 CFUM-UP, Physics Department, University of Minho, 4800-058 Guimarães, Portugal; nmgparreira@gmail.com (N.M.G.P.); martin.andritschky@fisica.uminho.pt (M.A.); 2 CEMMPRE—Centre for Mechanical, Department of Mechanical Engineering, Engineering Materials and Processes, University of Coimbra, Rua Luís Reis Santos, 3030-788 Coimbra, Portugal; todor.vuchkov@ipn.pt (T.V.); albano.cavaleiro@dem.uc.pt (A.C.); sandra.carvalho@dem.uc.pt (S.C.); 3 LED & MAT-IPN—Laboratory for Wear, Testing and Materials, Instituto Pedro Nunes, Rua Pedro Nunes, 3030-199 Coimbra, Portugal; 4 Engineering Department, KLC—Technical Plastics, 2430-021 Marinha Grande, Portugal; Jorge.Ferreira@klc.pt

**Keywords:** chromium coatings, reactive sputtering, decorative coatings, coatings on plastics

## Abstract

The present work aims to study the impact of O and N addition on Cr-sputtered coatings on plastic (polycarbonate, PC) used in automobile parts, as a promisor alternative for auto part metallization, while eliminating the usage of toxic hexavalent chromium. The coatings were deposited using DC magnetron sputtering from a single pure Cr target in a reactive atmosphere (N_2_ and/or O_2_). The deposition of the coatings was performed maintaining the total pressure constant and close to 1 Pa by tuning Ar pressure while reactive gases were added. The target current density was kept at *J_W_* = 20 mA·cm^−2^. Structural characterization revealed a mixture of α-Cr, δ-Cr, β-Cr_2_N, and CrN crystalline structures as well as amorphous oxides. The coating hardness ranged from 9 GPa for the CrON coating to 15 GPa for the CrN coating. All deposited coatings showed a particularly good interface adhesion; adjusting the amount of O and N made it possible to tune the optical properties of the Cr-based coatings as desired. The promising results open future industrialization of sputtered Cr-based coatings for automotive industries.

## 1. Introduction

Many objects found daily in automobile or decorative industries are metal-coated plastic parts that replace traditional metallic materials. The benefits of these metallized plastics are the combination of low density, flexibility, design versatility, and low production cost of the plastics, while maintaining the shiny finish, high reflectivity, and conductivity of metals [1,2]. Most of these parts are manufactured using injection molding and are subsequently metallized using electroplating and electroless methods [3]. However, these practices of electroplating nonconductive parts include the use of highly toxic hexavalent chromium during surface activation [4,5] or in the chrome plating itself [6]. Hence, the European Union created a Legislation Directive to reduce the use of this human health and environmentally hazardous compound [7], that drives the needs in industry to develop other methods for deposition of coatings on polymers.

Industry and researchers are now focused on finding new surface technologies based on more environmentally friendly processes to substitute chrome plating. Various processes offer themselves as alternatives to chromium plating. Chemical vapor deposition (CVD) and physical vapor deposition (PVD), which include sputtering techniques and thermal spraying (HVOF) that are used to alter the surface of different substrates with different coatings, depending on the application, for valves, decorative surfaces, or tools [8,9,10,11]. Magnetron sputtering is one such technique, which can be used on a wide range of available polymers, and has a reduced environmental impact and is becoming an increasingly attractive industrial process for polymer metallization, especially for the deposition of chromium nitride (CrN) films [1,2,3].

The most common polymers used in the automobile industry are acrylonitrile butadiene styrene (ABS), polycarbonate (PC), and PC/ABS blends, there is also an interest in fiber-glass reinforced polyamide (PA) [12]. Typically, PC can be used in a variety of optical and technical applications, and its demand is increasing year on year. It is widely used in optical data storage devices, bulletproof windows, and food packaging. Because of its good properties, such as optical transmittance, excellent thermal and flame resistance, high impact strength, and high stability to different environmental conditions, PC is used in a wide range of industrial applications, such as in the automotive industry [13].

The metallization of polymers with a thin metallic layer using magnetron sputtering started in 1994, by Grimberg et al. [14], who deposited TiN onto ABS and stated that, in order to coat the polymer with TiN, two conditions should be met: (i) adhesion must be assured, as Cu and Ni layers were added by electroplating and (ii) that the deposition must be performed at a low temperature to avoid polymer degradation. Respecting this last condition, the author kept the deposition temperature around 100–110 °C. Later, Sukwisute et al. [15] deposited CrN coatings on ABS substrates using a reactive DC magnetron to achieve a higher wear resistance for the ABS surface. In that study, a hardness of up to 9.6 GPa was reached, despite the occurrence of voids and cracks. Recently, Pedrosa et al. [16] studied the impact of reactive magnetron sputtering conditions on the metallization of Cr-N coating onto ABC substrates to reach desirable protective and decorative properties, comparable to those obtained traditionally by electroplating.

In the present study, the authors aim to develop a hexavalent chromium free coating for polymeric substrates to be used in the automobile industry as an alternative to electroplated chromium coatings. The coatings are deposited by reactive magnetron sputtering from a chromium target using N_2_ and O_2_ as reactive gases onto polycarbonate (PC) substrates. In addition to depositing a pure Cr layer, CrN and chromium oxynitrides were produced to improve the hardness and allow a variation of the decorative appearance [17]. Adding oxygen to CrN coatings provides a wide range of shades of grey without sacrificing wear and corrosion resistance [18,19,20]. Among the typical difficulties of the deposition of metallic coatings on polymers is adhesion; with that in mind, an interlayer was used and the impact of multilayers was also studied.

## 2. Materials and Methods

The coatings were deposited using DC reactive magnetron sputtering, using a home-made vacuum chamber, onto monocrystalline silicon wafers (100 P-type) supplied by SIEGERT WAFER GmbH (Aachen Germany), and polycarbonate (PC) (LEXAN ^TM^) samples were supplied by SABIC (Al-Jubail, Saudi Arabia). The PC substrates were coated with a UV-cured basecoat prior to metallization to improve both the adhesion and decorative appearance of the coating.

The samples were cleaned with isopropanol and then loaded into the deposition chamber. During the sputtering process, a single Cr target (99.5% purity) was used, Ar gas was used as the sputtering agent and reactive gases N_2_, O_2_ or a gas mix N_2_ + O_2_ (85% N_2_ and 15% O_2_) were added at different flow rates, as indicated in Table 1. During the depositions, no external heating was used to keep the deposition temperature as low as possible and a 3.5 rpm rotation was applied to the substrates. The base pressure in the chamber was approximately 1 × 10^−3^ Pa and the working pressure was kept at approximately 1 Pa. This is a relatively high pressure for reactive magnetron sputtering and was selected to reduce the energy of the species that bombard the substrates, therefore reducing the temperature increase during deposition. Before every deposition, an etching process was performed using a pulsed power source at 200 kHz, with a pulse width of 1536 ns and 400 mA current for 15 min. A chromium interlayer was deposited prior to coating for 50 s to enhance adhesion. The deposition time for each sample is also presented in Table 1. In essence, total deposition time was 360 s, except for the multilayer coatings which was 480 s. The multilayer coatings were deposited using similar conditions as indicated, with each layer being deposited for 60 s. The last layer of CrN/CrO was Cr_x_O_y_ and for CrN/CrON it was Cr_x_O_y_N_z_. No bias voltage was applied during deposition to protect the substrates.

The thickness and morphology of the coatings was analyzed using a NanoSEM FEI Nova 200, equipped with a Pegasus X4M for EDS chemical composition analysis. The structure of the coatings was analyzed by X-ray diffraction in a X′ Pert Pro MPD diffractometer operating with Cu Kα radiation (λ = 1.5406 Å at a grazing incidence angle of α = 3° and in a 2θ interval of 30–80°). Peak deconvolution was performed in Origin(Pro)9 (OriginLab Corporation, Northampton, MA, USA) using a Pseudo-Voigt function.

The hardness (H) and reduced Young’s modulus (Er) were measured by nanoindentation (Micro Materials Nano Test platform, Wrexham, UK)), with a Berkovich diamond pyramid indenter, applying a load of 3 mN to ensure that the indentation depth was less than 10% of the coating thickness. A total of 16 indentations were performed and the average was calculated.

The adhesion of the coatings on the PC substrates was tested using a cross-cut test following the ISO 2409 standard. Two sets of 11 cuts with a spacing of 1 mm were made perpendicular to each other, thus making a grid of 100 small blocks. Then, a standardized tape (Tesa^®^ 4657) was applied on the crosscut and pulled off with a constant force. The number of blocks removed was an indication of the adhesion, following the standard classification between 0 to 5, 0 being a perfect adhesion coating where the edges of the cuts are completely smooth and none of the squares of the lattice detached, and a classification of 5 corresponding to a coating that flaked along the edges of the cuts in large ribbons and/or squares that detached partly or wholly in a proportion higher than 65% of the tested area.

Dry sliding reciprocating tests were performed using the RTEC MFT-5000 platform (RTEC INSTRUMENTS, San Jose, CA, USA). Al_2_O_3_ balls with a diameter of 10 mm were used as counterparts. The normal load was set to 1 N, resulting in an initial contact stress of 37 MPa. The stroke was set to 2.2 mm. The reciprocating frequency was 5 Hz. The test duration was limited to 1 min, resulting in a total sliding distance of 1.21 m. For assessing the wear rate, an Alicona 3D optical profilometer was used was used. The specific wear rates (*K_i_*) were obtained using Equation (1), where ∆*V*_i_ is wear volume, *F_N_* is the normal load, and *L* the sliding distance [21,22]:

(1) $$K_{i}=\frac{∆V_{i}}{F_{N}\times L}$$

The color and reflectance of the coatings were measured using a Minolta CM-2600d portable spectrophotometer equipped with a 52-mm diameter integrating sphere, 3 pulsed xenon lamps, and a wavelength range of 400–700 nm. The color coordinates were measured in the CIELab-1976 color space at a viewing angle of 10° and using the primary illuminant D65 (specular component included—SCI).

## 3. Results and Discussion

### 3.1. Functional Characterization: Colour and Adhesion

As previously defined, these coatings were to be used decoratively in the automobile industry, in which color and adhesion are important and critical factors.

The color coordinates and reflectance of the films were measured to check if, and which of, the deposited coatings complied with end-user specifications. It is important to mention that color is not only dependent on the material composition, but is also related to surface roughness. Once this analysis was done for similar substrates, roughness impact could be excluded for analysis.

**Figure 1:**
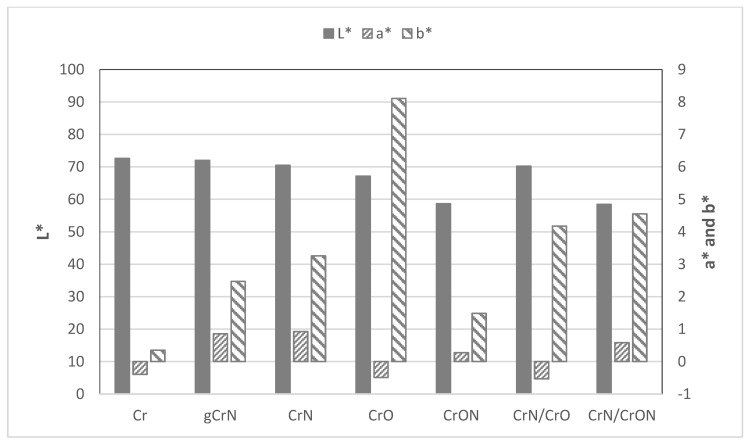
CIELAB color coordinates of the sputtered coatings onto PC substrates.

The color coordinates of the deposited coatings in the CIE L*a*b* system are shown in Figure 1. Note that L* is the brightness, where 0 = black and 100 = white; a* represents red and green on the positive and negative axes, respectively and b* represents yellow and blue on positive and negative axes, respectively [23].

Typically, with the increase of non-metallic elements, N or O, the brightness (L* coordinate) of the samples suffers a decrease [24]. With our samples, having up to 40 at.% non-metallic elements, coatings Cr, gCrN, CrN, and CrO, (see Table 1 for composition reference) showed similar brightness: close to 70. The color appearance differs, driven by the a* and b* coordinates, in particular the b* coordinate that increases from 0.5 for Cr to 8 for CrO coating gives it a yellow tonality.

The CrON coating is the one that showed a darker appearance, displaying a tonality of dark grey. Note that this coating was the only one with a higher non-metallic element (>50 at.%).

The multilayer coatings, CrN/CrO and CrN/CrON, did not exhibit major differences in color in comparison with the monolithic coatings. These coatings presented a tonality and color coordinates, in particular L*, ruled by the coating’s top layer, which was CrO for the CrN/CrO coating and CrON for the CrN/CrON coating.

**Figure 2:**
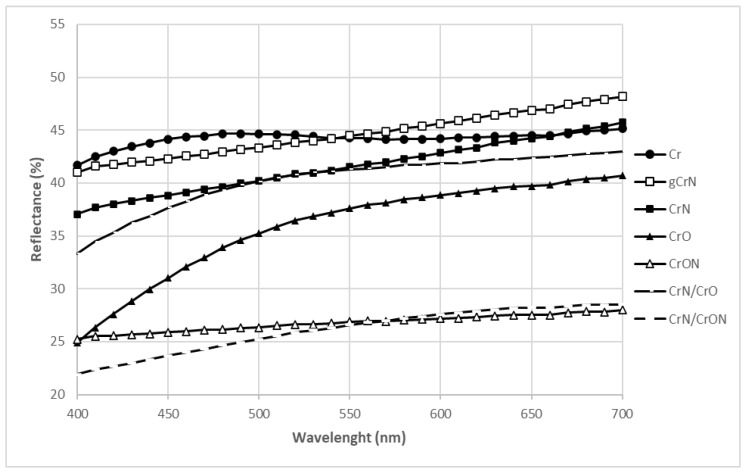
Reflectance evolution of the sputtered coatings deposited onto PC substrates.

The reflectance behavior, Figure 2, was consistent with the results presented by the brightness; in fact, Cr, gCrN, CrN, CrO, and CrN/CrO showed similar reflectance with a value between 35 and 50% in the range of 400 to 700 nm, and CrON and CrN/CrON presented a similar reflectance which was lower than the previous group with a reflectance value between 20 to 30% in the defined range.

**Figure 3:**
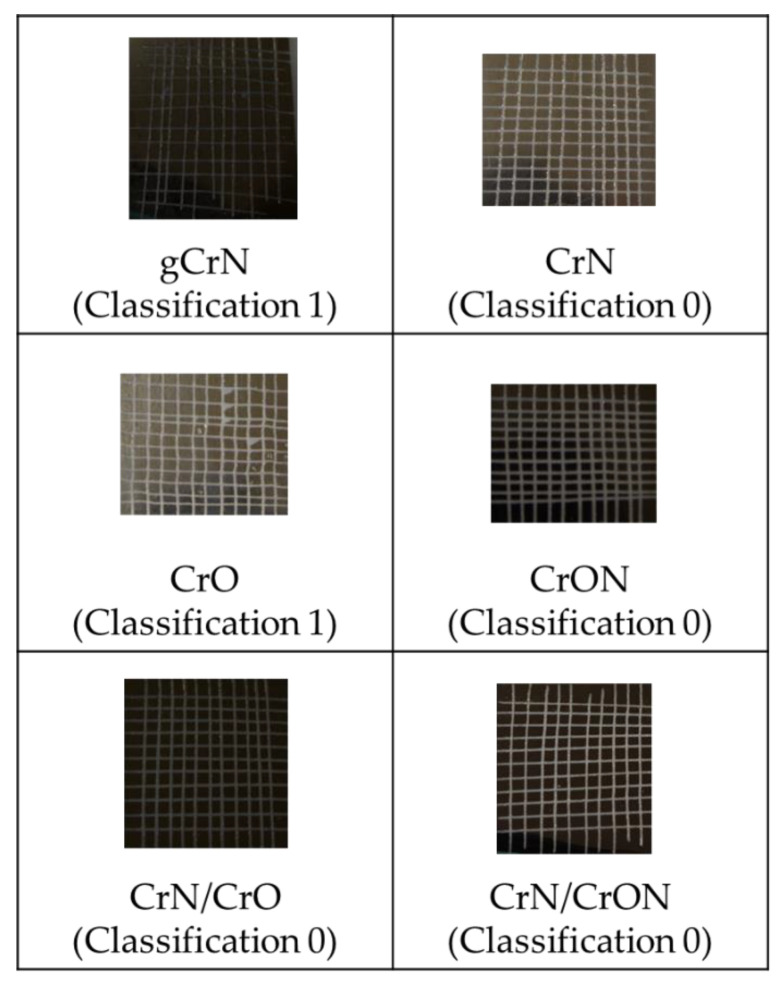
Adhesion behavior assessment of coatings deposited onto PC substrates.

Other important end-user specifications in the development of coatings onto polymers is to have good adhesion to the substrate. Therefore, the adhesion of the coatings was tested using the cross-cut tape test and the results are presented in Figure 3.

According to the scale defined by the standard, the adhesion of the deposited coatings was rated between 0 (perfect adhesion, where the edges of the cut were completely smooth and none of the squares of the grid were detached after removal of the tape) and 1. A special mention should be made regarding the gradient coating, gCrN, which showed a detachment of small flakes of the coating at the intersection of the cuts in an area smaller than 5%. From these results, the use of gradient CrN coatings seems to be uninteresting for further research. The CrO coating also presented a poor adhesion when compared to the other coatings. Color also played a part and in the case of end-user preference, as this composition/color is necessary to optimize adhesion by using a multilayer configuration that, as our results in Figure 3 point out, is promising.

In general, the adhesion of these coatings is very good for industrial applications and it is important to mention that the presence of the acrylate layer between the coating and the polymer substrate enhanced the adhesion.

### 3.2. Deposition Conditions and Basic Characterization

In the previous sections, we showed the possibility of tuning the color of Cr-based coatings deposited via sputtering onto polycarbonate substrates, and the good adhesion that we were able to achieve for most of these coatings. In this section, some of the fundamental characterizations (chemical composition, morphology, and structure) will be presented and discussed.

#### 3.2.1. Chemical Composition

The chemical composition for coatings deposited on Si-wafer substrates was measured using WDS, as shown in Table 1.

We observed a high amount of oxygen in of our all samples, even in the pure Cr coating. This can be explained by the fact that the deposition of the coatings occurred at a relatively high pressure, around 1 Pa, and that at these working pressures residual oxygen from the chamber can be incorporated into the films. It is also likely that the oxygen presence came from the degasification of the plastic substrates or from the epoxy layer that acted as a pre-coating.

It is worth mentioning that, in the chemical composition results presented for graded and multilayered growth, the stated composition is the combination of several different layers of composition, and not individual layers.

The individual results revealed that:

(1) gCrN and CrN coatings: For graded CrN coatings, gCrN, deposited with increasing nitrogen flux presented lower N content in comparison with the CrN coating. The 33 at.% N introduced in the CrN coating on a N_2_/Ar flow-rate ratio of 0.35 was in line with previous work by Mayhofer et al. [25];
(2) CrO coating: By adding O_2_ as the reactive gas inside the deposition chamber, it is possible to incorporate a significate amount of oxygen (30 at.% of O), even with a much lower gas flow than for N_2_-presence coatings, which is explained by the higher reactivity of oxygen to Cr [26];
(3) CrON coating: When a gas mixture of 85% N_2_ + 15% O_2_ was added to the atmosphere for the CrON coating, the number of reactive species inside the deposition chamber became higher and this led to the highest non-metallic elements amount being added to the coatings in the present study (~21 at.% of O and ~33 at.% of N). Note that, proportionally, the amount of O in the coating was higher than the amount of O_2_ in the gas mixture (85% N_2_ and 15% O_2_) which can be explained by the higher affinity of O to Cr than N to Cr, as indicated by the standard molar enthalpy of formation of Cr_2_O_3_ and CrN: Δ_f_*H*° (Cr_2_O_3_) = −1139.7 kJ/mol and Δ_f_*H*° (CrN) = −117.2 kJ/mol [26], also observed in previous studies from our research team on oxynitrides coatings [27];
(4) CrN/CrO and CrN/CrON coatings: In the case of multilayered coatings, CrN/CrO and CrN/CrON, the chemical composition measured using this method showed a value similar to the average of the chemical composition of individual layers (CrN and CrO or CrON), which indicated that the analytical method had been influenced by the several layers of depth.

**Figure 4:**
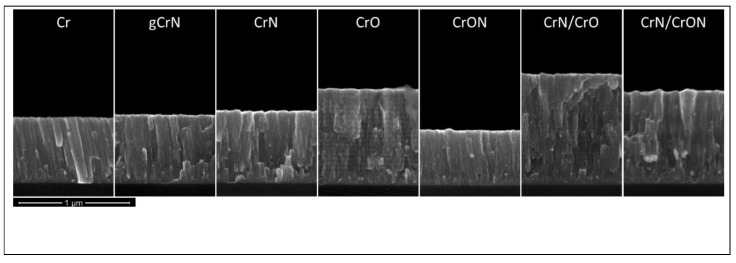
Scanning electron cross-section micrographs of the coatings deposited on silicon.

The thickness of the coatings was measured by cross-section analysis of SEM micrographs on silicon and after the deposition rate was calculated. These results are shown in Table 1 and Figure 4 as a function of the deposition conditions.

The coating thickness ranged between 500 nm and 1020 nm and the deposition rate varied between 1.4 and 2.4 nm/s depending on the deposition conditions. Under the defined conditions, the deposition rate of pure Cr coatings was close to 1.7 nm/s and for gCrN and CrN coatings it showed a value of 1.8 nm/s. This clearly showed that the deposition was done in a metallic mode, even up to 33 at. % addition of N. Combining the deposition rate value and the chemical composition, this clearly shows that the deposition is still far from the full poisoning of the target and so the stoichiometric composition of CrN was not reached [28]. For samples deposited with oxygen as the reactive gas (CrO coating), the thickness increased and the deposition ratio reached 2.4 nm/s. This is typical behavior of Cr-O coatings when deposition conditions remain in the metallic mode and it is a consequence of the incorporation of O atoms in the Cr lattice, which leads to a structural change with the formation of new phases having different atomic arrangements, which give rise to a lower specific molecular weight and consequently to an increase in the coating volume and final coating thickness. This behavior was confirmed by Rothhaar et al. [29] and also observed in Mo-O [30] and W-O systems [31].

Multilayer coating of CrN/CrO and CrN/CrON showed a deposition ratio that was very close to the average deposition ratio of CrN and CrO or CrON, respectively. In fact, as will be presented in the discussion on morphology, these multilayer coatings showed two clear visible layers (see Figure 4) for CrN and CrO or CrON, respectively.

#### 3.2.2. Morphology

The scanning electron cross-section morphology of the deposited coatings is shown in Figure 4.

Cr coatings showed a well-defined columnar structure, and the same was observed for the gCrN and CrN coatings. In fact, this is typical behavior for coatings deposited up to 1 Pa (high pressure) without external heating (low deposition temperature) [32,33]. For the CrON coating, a columnar structure was still observed, but was less pronounced.

**Figure 5:**
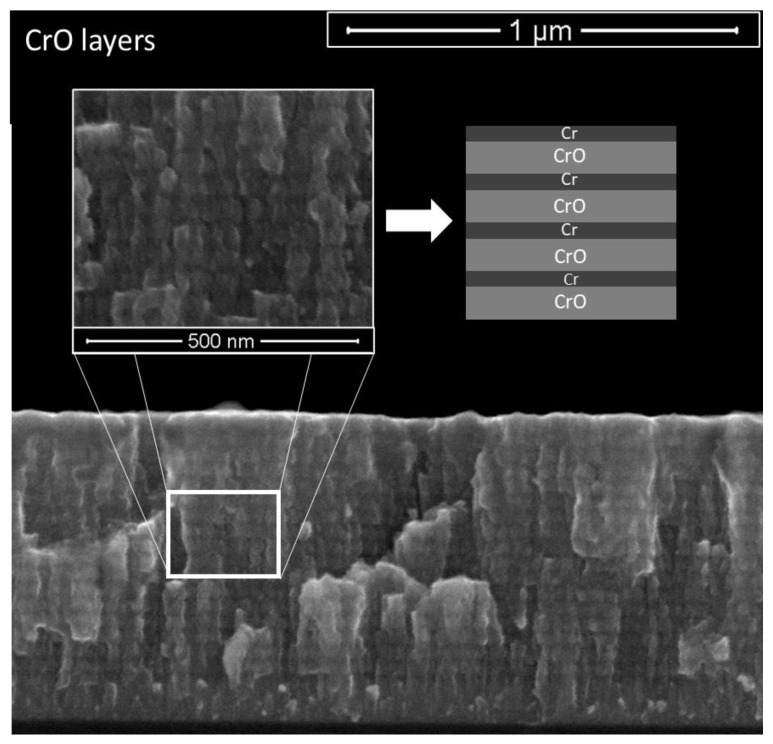
Cross-section SEM micrograph of the CrO coatings. We observed a multilayer-like structure with charged and uncharged layers, corresponding to layers deficient and rich in O, respectively.

The obtained CrO coating observed in the SEM micrographs showed an interesting phenomenon, which was a multilayer growth (Figure 5). This multilayer morphology can be explained by the deposition conditions, namely the depositions were carried out using only one Cr target in a non-poisoned mode (transition zone) and the substrate holder rotated at 3.5 rpm. Therefore, the formation of a differentiated plasma surrounding the substrate holder could be expected. When the samples were facing the Cr target, a fresh metallic Cr layer was deposited, and the amount of oxygen was reduced due to the high flux of Cr atoms coming from the target. In the remaining time, when the samples were turned around, the oxidative plasma environment (Ar + O_2_) promoted the oxidation and adsorption of oxygen by the films, which resulted in an increase in oxygen on the surface of the Cr layer. This phenomenon is clear in Figure 5, where charged (brighter) and uncharged (darker) layers are visible, which correspond to O-rich and O-deficient layers, respectively. This behavior was also found in other similar systems in previous studies from our research group [34].

**Figure 6:**
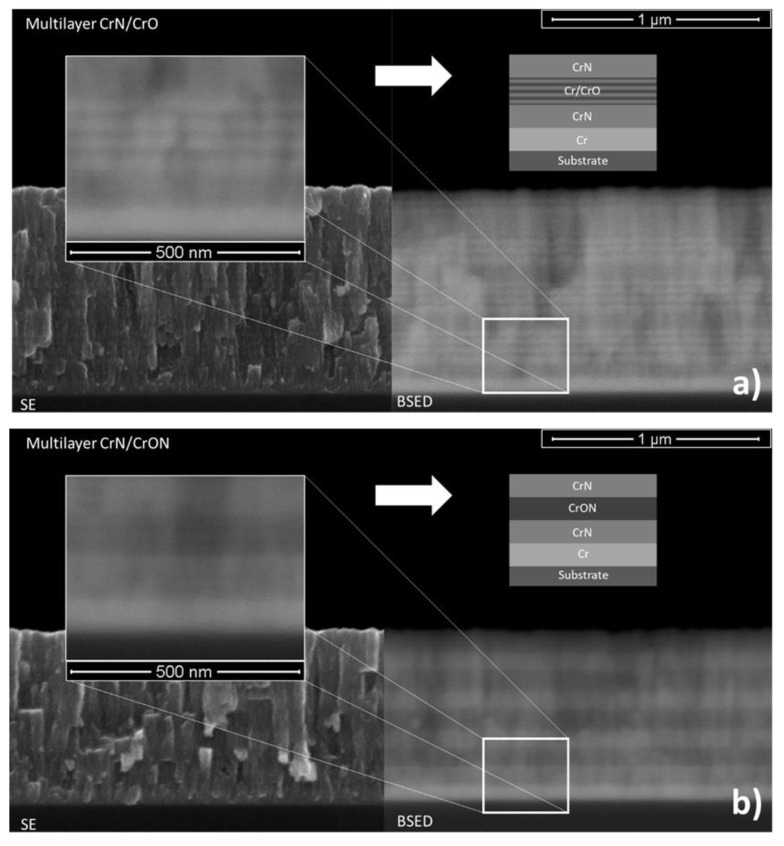
Cross-section SEM micrography in secondary electron (**left**) and backscattered electron modes (**right**) for (**a**) CrN/CrO and (**b**) CrN/CrON multilayer coatings.

The cross-section SEM images of the multilayer coatings in secondary electron (SE) and backscattered electron (BSED) modes are shown in Figure 6. The CrN/CrO coating, as shown in Figure 6a, shows, bands similar to CrO interrupted by a darker “solid” band in the SE mode. This is even more evident in the BSE mode, where brighter and darker areas are more contrasting. In the BSED mode, lighter areas correspond to heavier elements or metallic ones [35], meaning that from the bottom-up, one can see a Cr interlayer, CrN layer, and a layer of Cr-O that, in fact, are four bilayers of Cr/CrO due to the phenomenon described earlier for CrO coatings. This is repeated four times, which corresponds to the deposition of 60 s of each layer for a total time of 480 s, as per Table 1.

Regarding CrN/CrON coatings, a similar but not so evident behavior was observed. On the left of Figure 6b, using the BSED mode (right), a Cr interlayer was present at the bottom and, after, the 4 bilayers of CrN/CrON were observed, as expected.

#### 3.2.3. Structural Characterization

**Figure 7:**
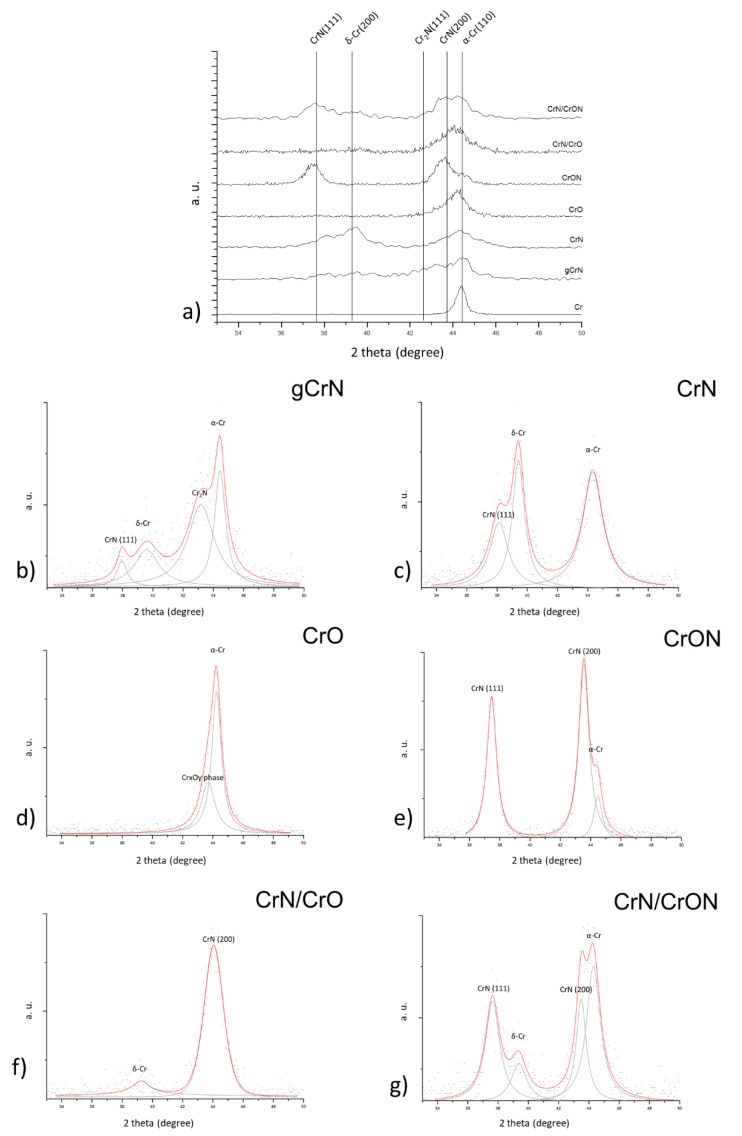
XRD patterns of the deposited coatings, (**a**) plot of all XRD specta for comparison purposes. Individual detailed analysis of the XRD spectra of the different coatings: (**b**) gCrN; (**c**) CrN; (**d**) CrO; (**e**) CrON; (**f**) CrN/CrO and (**g**) CrN/CrON.

The crystallographic structure was evaluated using X-ray diffraction (XRD) and the results are shown in Figure 7a for all deposited coatings.

Starting from the monolayer metallic chromium coating, Cr, an intense reflection close to 2θ = 44.4° corresponding to the (110) plane was observed for the b.c.c α-Cr, according to ICCD card No. 01-085-1335.

For the CrN coating, deposited with a constant partial pressure of nitrogen, we observed the (111) peak from CrN phase according to ICCD card No. 01-076-2494. Close to 2θ ≈ 39.4° a strong peak is visible, which does not correspond to any known phase of the binary Cr-N system, but can correspond to the reflection of the (200) plane of the metastable Cr phase, δ-Cr, according to ICDD card No. 00-019-0323. This phase is a A15 crystallographic structure, may occur in thin films, and is stabilized by impurities like oxygen or nitrogen [36,37,38,39]. The presence of δ-Cr is also found in previous multilayer Cr/Cr-N studies [40,41]. To complete the phase composition of the CrN diffractogram, is not possible to exclude the presence of the b.c.c α-Cr phase close to 2θ ≈ 44.3°, since these coatings presented a sub-stoichiometric composition (see Table 1) and oxygen was present as an impurity; thus, a wider peak. Complete analysis of the XRD pattern of CrN (Figure 7c) showed that the coating was composed of a mixture of α-Cr, δ-Cr and CrN phases.

The gCrN coating was produced by a gradual increase in the nitrogen flow during deposition, generating a coating with a richer N content from the bottom of the coating, starting from pure Cr, to a CrN composition at the top. Using XRD results it is possible to identify a broad peak in the range between 42° and 44.5°, which can be assigned to a mixture of different phases, ranging from Cr to CrN, in agreement with the graded composition. It was observed the b.c.c α-Cr and reflection close to 2θ ≈ 43.2° which correspond to the (−1 −1 1) plane from the hexagonal Cr_2_N phase, according to ICDD card No. 01-079-2159. We also observed the CrN crystallographic phases. By applying deconvolution of the gCrN XRD pattern (Figure 7b) it is possible to also observe broad peaks with low intensity corresponding to CrN, close to 2θ ≈ 37.6° and the δ-Cr peak at 2θ ≈ 39.4°. So, the crystallographic phases present in the gCrN coating are primarily α-Cr (from the initial phase of coating deposition) and hexagonal Cr_2_N in the bulk of the coating with traces similar to the CrN coating phases, which are present on the top of the coating with a lower XRD intensity.

The XRD pattern of the CrO coating showed one major diffraction peak between 2θ ≈ 42° and 47°. By peak deconvolution, see Figure 7d, the observed peak is composed of a main peak attributed to α-Cr and a broad superimposed peak that can be attributed to chromium oxide phases. From the ICDD database on the Cr-O system, this peak at 2θ ≈ 43.6° can be attributed to the Cr_3_O phase (ICDD card No. 01-072-0528) or the CrO_0.87_ phase (ICDD card No. 01-078-0722) and both are cubic structures. Note that the described δ-Cr, A15 structure is an isomorphic structure with Cr_3_O and with (210) preferential orientation. As mentioned in the morphological analysis section, this CrO coating exhibited a multilayer structure with Cr-rich zones and O-containing zones, so it was possible to have these structures together in a nano-arrangement. We also noticed a small peak shift to the left (higher diffraction angles) indicating tensile stresses in the coating, and, when compared to pure Cr coatings, the crystallite size went from 16.8 nm to 7.9 nm in the CrO coating, calculated using Scherrer’s equation [42].

The XRD pattern of the coating deposited with the mixture of O_2_ + N_2_ gas flow, coating CrON, see Figure 7e, exhibited both (111) and (200) reflections of the CrN cubic structure. Close to 2θ ≈ 44.5°, there was a superimposed peak that can be attributed to α-Cr or δ-Cr with a preferential orientation (210), or to a rich oxygen phase, such as Cr_3_O or CrO_0.87_. Even with this combination of phases, there is excess oxygen in the coating composition. It is possible to have some oxygen in a solid solution, but the solubility of oxygen in CrN is very limited [43] and the stoichiometric combination was most probably achieved by the presence of amorphous Cr_2_O_3_ in the coating structure which is the most stable phase of the Cr-O system, even if it is not visible by XRD it contributes to the mechanical properties of the coatings (see next section). From the literature, limited works have been done on CrON systems using reactive magnetron sputtering. The work by Collard et al. [43], which focused on adding N starting from a Cr_2_O_3_ structure or adding O to a CrN structure, allowed the study of solubility of N and O on oxides and nitrides, respectively, by replacement of other non-metallic atoms or interstitial positions. In Collard’s study there was no information concerning chemical composition, only flow rates were presented and it was shown that above a ratio of 5 sccm oxygen to 15 sccm nitrogen, a coating only showed XRD reflections from oxide, mainly Cr_2_O_3_-eskolaite. The gas mixture used on the present study, 85% N2 and 15% O_2_ by volume, had a lower ratio of oxygen that in Collard’s study, so a simple model of the phase mixture of this coating could be a combination of CrN(O) nanocrystalline phases in a matrix of amorphous Cr_2_O_3_ and α-Cr or (210) δ-Cr or Cr_3_O.

Concerning multilayer coatings, CrN/CrO and CrN/CrON, X-rays penetrate and give information on several layers of the coatings. What is observed from the XRD pattern of these coatings (Figure 7f,g, respectively) is a mixture of the individual single phases, CrN, CrO or CrON. Looking in detail at CrN/CrON coatings, the XRD pattern shows a mixture of α-Cr (110) plan, δ-Cr, in particular, the (200) reflection and CrN (111) and (200) plane all present in the CrN layer and are superimposed by CrN reflections that are also present in the CrON layer. The impact of this multilayer arrangement will have an impact on the mechanical properties of the coatings.

### 3.3. Mechanical and Tribological Characterization

#### 3.3.1. Mechanical Characterization

**Figure 8:**
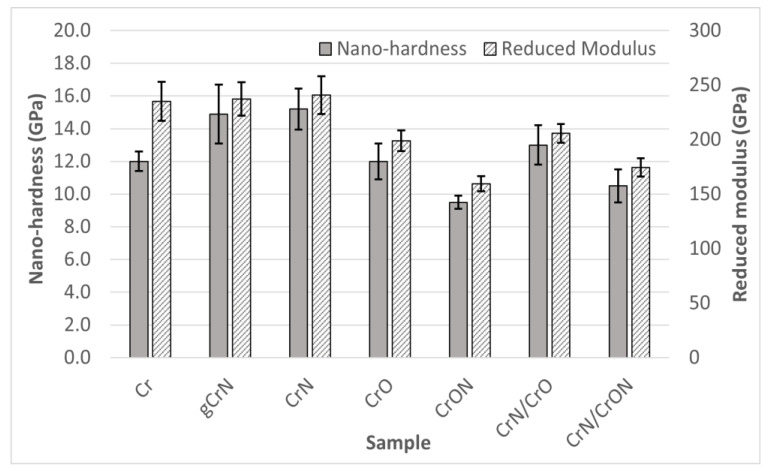
Nano-hardness and Young’s modulus of the coatings deposited onto Si.

Having good mechanical properties is vital for potential industrial applications of the coatings. In Figure 8, hardness (H) and Young’s modulus (Er) as measured by nanoindentation are presented.

The Cr coating has a hardness value of 12 GPa and a Young’s modulus of 235 GPa, values that are relatively similar to those reported in the literature [25,44], which shows values close to 10 GPa and 240 Gpa for H and Er, respectively. The relative higher hardness is even more noteworthy keeping in mind the columnar structure of the coatings, as viewed in SEM micrography (Figure 1). Although not presented in this study, we noticed that Cr coating presented a relatively high compressive residual stress, which can explain the higher hardness. In studies where negative bias was applied, for example by Forniés et al. [45], a hardness of 12 GPa was also observed.

For gCrN and CrN coatings, as explained in the chemical composition discussion, both have similar N contents (~35 at.%) on the top of the coating, the structure of the films is composed by similar phases, and thus the hardness and Young’s modulus show similar values at ~15 GPa and ~240 GPa, respectively. This hardness value is in line with previous works [46], showing that Cr_2_N + CrN or pure CrN structures have a hardness close to 15 GPa. A *Er* value of 240 GPa was also reported by Elangovan [47] in past studies.

The deposited CrO coating exhibits a hardness value of 12 GPa and a Young’s modulus of 199 GPa. These values are in the range of values presented by Pang [48] and Fernandes [49], in particular when deposition happens in the metallic mode, with a sub-stoichiometric composition and an amorphous structure. When associating the chemical composition, X-ray diffraction and hardness results of our CrO coating with the work of Barshilia [50], in which a bigger range of Cr-O composition was studied, it reinforces the explanation that our coating is far from a stoichiometric Cr_2_O_3_ coating, which has a reported hardness of 7 GPa.

The CrON coating exhibited lower hardness results in this series with 9.5 GPa. Note that this was the coating with a more pronounced CrN crystalline structure, despite the relative high amount of oxygen (18~22 at.%) (see Figure 7e). So, the presence of oxygen stabilizes the formation of the CrN crystal phase, being that the oxygen atoms in interstitial positions broadened the crystallographic peaks and/or replaced some nitrogen atoms in the CrN structure. In addition, it could be possible that some Cr-O amorphous phases existed and thus created a nanocomposite of CrN nanograins in a Cr-O amorphous matrix (CrN@Cr-O). Some of the pioneering works on the Cr-O-N system from Collard et al. [43] showed a limited incorporation of oxygen into the CrN structure. In fact, according to Collard et al., when incorporating oxygen into CrN, a nanocrystalline structure with an amorphous fraction evolves gradually into a Cr_2_O_3_-type structure. In previous work by the authors on a W-O-N system [39], the rule of mixtures could be used to predict the hardness of the coatings. Assuming a nanocomposite model CrN @ Cr-O matrix, the hardness of these coatings could be calculated based on the rule of mixtures, using the hardness of CrN as 15 GPa (from this work) and the hardness of Cr_2_O_3_ of 7 GPa [50], the calculated hardness was 12 GPa, estimating 70% of CrN and 30% of Cr_2_O_3_, but also taking in consideration the material density, the volume of the CrN phase being 62% and Cr_2_O_3_ being 38%. These calculated values are clearly more than those of the experimental data. Other works on Cr-O-N systems using cathodic arc evaporation found a higher hardness, close to 14 GPa [44], attributing that value to the limited existence of microstrains in the crystal structure, resulting in high compressive residual stress [51]; however, using the arc evaporation method, the Cr_2_O_3_ hardness was higher since the coatings showed a much higher crystallinity than films with similar compositions produced by sputtering.

Concerning the mechanical properties of the multilayer coatings, CrN/CrO and CrN/CrON, it is important to mention that these multilayers have a relative long period, Λ, between 140 and 250 nm, so an enhancement in mechanical properties due to interface influence it is not expected (Hall and Petch [52] and Lehoczky’s [53] theories) yet a behavior close to the rule of mixtures is predictable. The hardness value of CrN/CrO was 13 GPa, if taking the rule of mixture into consideration for the hardness of CrN and CrO it should result in 13.6 GPa. Using the same approach, the coating CrN/CrON, showed a hardness of 10.5 GPa when calculated by the rule of mixtures and the average between the CrN and CrON was 12.3 GPa. We always observed a lower hardness value than the average of the individual layers, even if recalculated based on the layer volume, as observed in Figure 5 for CrO, with bigger layers and a lower hardness. If the volume weight average was used, the hardness value should be lower but remain higher than the real measured value. As far as we know, there are no studies in the literature concerning CrN/CrO nor CrN/CrON systems. There are some Cr/CrN studies, even with long multilayer periods, in which some similarities can be found, and we can extrapolate information assuming Cr as the softer layer and CrN as the hard layer. Marulanda et al. [54] observed a softening of the coating as the layer period thickness increased, but there is a lack of information for the reference of monolithic coatings under the same deposition conditions. Kot et al. [55] mentioned that coatings with a 1000 nm period show hardness and modulus values close to those of pure Cr, near the softer layer, whereas the coatings with a layer period of 250 and 500 nm had a hardness and modulus similar to the harder layer, CrN, and this is attributed to the Hall-Petch effect. In Kot’s work, they found that hardness of a multilayer increases when the layer period decreases, although this study was done for laser cladding and we did not observe a similar result in our study. Another study from Arias et al. [56] also explained the higher hardness of their multilayers with a long period (between 200 to 500 nm) resorting to the Hall-Petch effect, concluding that Cr/CrN with long-period multilayers have a hardness higher than CrN. Ultimately, different behaviors can be observed. Previously, the authors of this paper worked on a different multilayer system with a long layer period, W/W-O [57], and in that case, W layers were harder and W-O were softer layers; the overall hardness of the coatings also did not follow the rule of mixtures, but tended to be closer to the hardness of the soft layer, W-O.

#### 3.3.2. Tribological Characterization by Dry Sliding Reciprocating Tests

In the automotive industry, some parts in which decorative coatings are applied, are either under heavy human contact (door handles and knobs) or suffer some sort of abrasive aggression from the surrounding environment. To find out how these coatings behave, samples were tested using a dry sliding reciprocating test against an Al_2_O_3_ ball that the authors consider a good testing counterpart to represent sand or other environmental debris that can damage surfaces.

**Figure 9:**
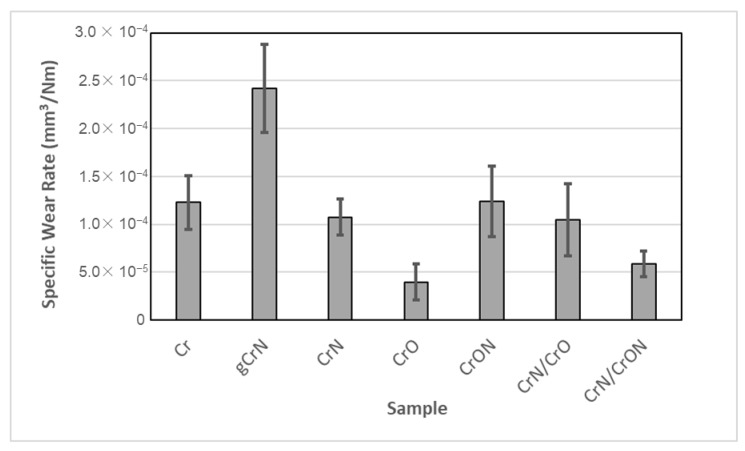
Specific wear rate of the coatings tested using dry sliding reciprocating tests.

The dry sliding reciprocating tests of the coatings are presented in Figure 9. The results showed a similar specific wear rate for most of the coatings of around 1 × 10^−4^ mm^3^/Nm, with the exception of gCrN, CrO, and CrN/CrON. It is important to mention that the tested coatings were deposited onto polycarbonate, a polymeric substrate, and a limited number of cycles and load were applied, since our goal was to assess the durability of the coatings.

As shown, under these test conditions, coatings Cr and CrN presented similar wear rates, which is different from other tribological studies. To the best of the authors’ knowledge, there are no tribological characterizations of Cr and CrN coatings using dry sliding reciprocating tests, only tribological studies using a pin-on-disc; in those cases, the authors showed a correlation between hardness and tribological behaviour, with CrN coatings exhibiting a lower wear rate than Cr coatings (as an example, the work of He et al. [58]). Another coating with a relatively high hardness is gCrN, which presents the worst behavior in this series, and can be attributed to the lower adhesion of the gCrN coating, as shown in Figure 3.

The CrO coating is highlighted in this study due to it having the best tribological behaviour, in spite of its relative lower hardness and classification of 1 in the adhesion test. This result is in line with that of a previous study from Urgen et al. [59], which showed that a CrO coating presents lower wear in comparison with CrN or CrON coatings. The multilayer coatings CrN/CrO and CrN/CrON, especially the latter, present a good wear resistance in the dry sliding test, which is an expected result, consistent with the literature, where the multilayer coatings present a better wear resistance behavior than the individual layers [56].

We considered that all the coatings presented in this study are good candidates for metallization coatings onto plastic, with the exception of the gCrN coating, which exhibited a high wear rate, limiting its use for the intended applications of this study.

## 4. Conclusions

The results presented in this work are part of a study to develop environmentally friendly decorative Cr-based coatings for the metallization of automotive parts to replace hexavalent chromium-plated solutions.

Coatings were deposited by DC reactive magnetron sputtering from a single pure Cr target in a reactive atmosphere (N_2_ or O_2_ or a mixture of the two gases). In the defined conditions, it was possible to incorporate up to 32 at.% oxygen (in the case of the “pure” CrO coating) and 36 at.% N in the case of the CrN coating. The deposition rate varied between 1.4 nm/s for the CrON coating and 2.4 nm/s in the case of the CrO. It is worth mentioning that the CrO coating exhibited a higher deposition rate than the nitrogen-containing ones.

The morphology of the coating was columnar for the Cr and CrN coatings, type T according to Thornton model for the CrON coating, and CrO presented a multilayer effect due to the high deposition rate of the oxide phase and the fact that the deposition was done under rotation. Concerning the structure, depending of the composition, the coatings were b.c.c α-Cr, δ-Cr (a metastable A15 cubic structure), the hexagonal β-Cr_2_N and cubic CrN. Note that it was not possible to directly detect oxide phases, but once the Cr_3_O and CrO_0.87_ phases with the reflection places were superimposed in the (110) plane of α-Cr, it was not possible to exclude (by XRD) the presence of these phases. We did not observe an oxide phase using XRD in the CrON coating, even at 22 at.% O and only a reflection of CrN was observed, leading us to the idea that the coating was a nanocomposite with small CrN grains and with some oxygen in the solution in an amorphous oxide phase.

The mechanical properties of the coatings were measured using nanoindentation and showed a range of hardness between 9.4 GPa and 15.2 GPa for CrON and CrN coatings, respectively.

The adhesion of the coatings onto PC substrates was measured using a cross-cut tape test (using the ISO 2409 standard) and all coatings presented excellent adhesion. Exception must be made for the gradient coating gCrN and CrO coating, which showed detachments of small flakes in the intersection of the cuts in an area smaller than 5%.

In dry sliding test, all coatings, with the exception of gCrN, were proven to be good candidates for automotive applications, showing wear rates compatible with the industry standards.

The color and reflectance of the coatings were measured by spectrophotometry and all coatings exhibited a grey tonality. Different brightness of coatings were obtained by changing the chemical composition, allowing the exploration of a range of grey tones, highly desired in the automotive industry.

Focusing our study on the Cr-O-N system for use as decorative coatings, we already defined some limits in terms of chemical compositions of coatings designed to enhance the performance of decorative coatings. Further studies concerning corrosion behavior and the impact of film thickness in future industrialization of sputtered Cr-based coatings in the automotive industry are underway.

## Figures and Tables

**Table 1:** Deposition conditions of the coatings (gas flow and deposition times), deposition rate and chemical composition characterization of the deposited coatings.

Coating	Gas Flow (sccm)				Deposition Time (s)		Thickness	Deposition Rate	Chemical Composition (at.%)		
	Ar	N_2_	O_2_	N_2_ + O_2_	Interlayer	Coating	(nm)	(nm/s)	Cr	O	N
Cr	100					360	603	1.7	92	8	-
gCrN	70	5→25				360	629	1.8	66	6	28
CrN	70	25			50	310	638	1.8	60	8	32
CrO	100		15		50	310	862	2.4	69	31	-
CrON	70			40	50	310	497	1.4	46	21	33
CrN/CrO	70	25	15		50	480	1020	1.9	63	25	12
CrN/CrON	70	25		40	50	480	858	1.6	49	18	33

## Data Availability

The data presented in this study are available on request from the corresponding author.
